# Growth patterns and clinical outcomes in association with breastfeeding duration in HIV exposed and unexposed infants: a cohort study in KwaZulu Natal, South Africa

**DOI:** 10.1186/s12887-021-02662-8

**Published:** 2021-04-19

**Authors:** Larisha Pillay, Dhayendre Moodley, Lynda Marie Emel, Ntombifikile Maureen Nkwanyana, Kimesh Naidoo

**Affiliations:** 1 Department of Paediatrics and Child Health, School of Clinical Medicine, University of KwaZulu-Natal, 719 Umbilo Road, Congella, 4013 South Africa; 2 Department of Obstetrics and Gynaecology, School of Clinical Medicine, University of KwaZulu-Natal, 719 Umbilo Road, Congella, 4013 South Africa; 3 Biostatistics, Bioinformatics, and Epidemiology/VIDD, Fred Hutchinson Cancer Research Center, Seattle, WA USA; 4 Discipline of Public Health Medicine, College of Health Sciences, University of KwaZulu-Natal, Glenwood, South Africa

**Keywords:** HIV, Infants, Breastfeeding, Growth trajectories, Clinical outcomes

## Abstract

**Background:**

Exclusive breastfeeding for 6 months and breastfeeding with complementary feeds until 12 months for HIV exposed and uninfected (HEU) infants or 24 months for HIV unexposed (HU) infants is the current World Health Organisation (WHO) recommendation for low and middle income countries (LMICs) to improve clinical outcomes and growth trajectories in infants. In a post-hoc evaluation of HEU and HU cohorts, we examine growth patterns and clinical outcomes in the first 9 months of infancy in association with breastfeeding duration.

**Methods:**

Two cohorts of infants, HEU and HU from a low-socioeconomic township in South Africa, were evaluated from birth until 9 months of age. Clinical, anthropometric and infant feeding data were analysed. Standard descriptive statistics and regression analysis were performed to determine the effect of HIV exposure and breastfeeding duration on growth and clinical outcomes.

**Results:**

Included in this secondary analysis were 123 HEU and 157 HU infants breastfed for a median of 26 and 14 weeks respectively. Median WLZ score was significantly (*p* < 0.001) lower in HEU than HU infants at 3, 6 and 9 months (− 0.19 vs 2.09; − 0.81 vs 0.28; 0.05 vs 0.97 respectively). The median LAZ score was significantly lower among HU infants at 3 and 6 months (− 1.63 vs 0.91, *p* < 0.001; − 0.37 vs 0.51, *p* < 0.01) and a significantly higher proportion of HU was classified as stunted (LAZ < -2SD) at 3 and 6 months (3.9% vs 44.9%, *p* < 0.001; 4.8% vs 20.9%, p < 0.001 respectively) independent of breastfeeding duration. A higher proportion of HEU infants experienced one or more episodes of skin rash (44.5% vs 12.8%) and upper respiratory tract infection (URTI) (30.1% vs 10.9%) (*p* < 0.0001). In a multivariable analysis, the odds of occurrence of wasting, skin rash, URTI or any clinical adverse event in HEU infants were 2.86, 7.06, 3.01 and 8.89 times higher than HU infants after adjusting for breastfeeding duration.

**Conclusion:**

Our study has generated additional evidence that HEU infants are at substantial risk of infectious morbidity and decreased growth trajectories however we have further demonstrated that these adverse outcomes were independent of breastfeeding duration.

## Background

UNAIDS reports more than 60% decline in new perinatal HIV infections in 2018 associated with the universal coverage of antiretroviral treatment in antenatal clinics worldwide [1]. South Africa (SA) has an estimated 3.5 million HIV exposed uninfected (HEU) children, the highest recorded in the world [2]. This is attributed to one of the most successful Prevention of Mother-to-Child Transmission (PMTCT) programmes in Sub-Saharan Africa (SSA) with universal combination antiretroviral treatment (cART) for all pregnant and breastfeeding women living with HIV, safe breastfeeding guidance for 12 months and repeat HIV testing during pregnancy and breastfeeding to identify new HIV infections and to allow for early commencement of cART [3, 4].

Despite the reduction in perinatal HIV infections and HIV-associated morbidity and mortality in the current ART era, the persistently high rate of gastroenteritis, acute respiratory infections and malnutrition in children in general in SSA is of increasing concern [5–7]. The rates of stunting, severe acute malnutrition and moderate acute malnutrition remain high in hospital admissions of HIV infected and HIV unexposed children [8].

Breastfeeding has long been established as the best form of adequate nutrition in infants to reduce the risk of childhood morbidity and mortality [9, 10]. The World Health Organisation (WHO) has made substantial investments towards promoting exclusive breastfeeding for six months and continued breastfeeding for up to two years [11]. Much attention, however, has been given to promoting and monitoring breastfeeding among WLHIV. There is limited evidence of feeding practices and infant wellbeing among WNLHIV. In a pooled analysis of 21 clinical trials involving over 19,000 HEU infants in SSA and Asia, half of the infant deaths occurred before three months of age [12]. While mothers not receiving cART for life contributed to almost 50% of infant deaths, never breastfeeding contributed to 10.8% of infant deaths. Current WHO infant feeding recommendations for HIV exposed infants in low- and middle-income countries (LMICs) are exclusive breastfeeding for six months and continued breastfeeding with complementary feeds until 12 months while mothers are virally suppressed on cART. For WNLHIV, it was recommended that infants be exclusively breastfed for six months but a longer duration of breastfeeding with complementary feeds thereafter until 24 months [11]. Two recent studies in SA reported short breastfeeding duration among women in general regardless of their HIV status [13, 14].

With the high rates of HIV infection in childbearing women in SSA and SA, in particular, there has been widespread use of antiretrovirals for treatment, prevention of mother-to-child transmission (PMTCT) and infant prophylaxis. The opportunity to assess the growth of infants and rates of malnutrition and childhood infections in infants born to mothers not exposed to antiretroviral drugs is becoming rare [15, 16].

Our study aimed at describing the growth patterns and clinical outcomes in two cohorts of infants born to WLHIV and WNLHIV and living in the same geographical and socioeconomic context. In a secondary analysis of data, we report the incidence of respiratory infections, rash or skin infections, diarrhoea, malnutrition and growth faltering in the first six to nine months of infancy in association with breastfeeding duration.

## Methods

This is a secondary analysis of two cohorts of infants born to WLHIV and WNLHIV residing in Umlazi, a peri-urban low-socioeconomic township in South Africa. Between 2007 and 2009, HIV exposed and uninfected (HEU) infants were enrolled in a multi-centred randomised placebo-controlled trial (HPTN046) designed to investigate the efficacy of extended Nevirapine prophylaxis in preventing breastfeeding transmission of HIV-1 [17]. For the secondary analysis, we selected infants who were enrolled in Umlazi and who did not receive NVP prophylaxis, nor did their mothers receive cART during pregnancy and breastfeeding. Between 2017 and 2018, pregnant women without HIV were enrolled in an observational cohort study (CAP088) designed to determine the incidence of HIV during pregnancy and breastfeeding [18].

Infants in the HPTN046 study were enrolled within seven days of birth if they tested negative with HIV DNA PCR, have a birthweight at least 2000 g and be able to breastfeed. Gestational age was not determined. Clinical, anthropometric and infant feeding assessments were conducted by research nurses and clinicians at two, five, six and eight weeks and three, four, five, six, nine, 12 and 18 months. In the CAP088 study, clinical, anthropometric and infant feeding assessments were conducted and documented in the Road-to-Health Card by primary health care nurses as per the IMCI guidelines at three, six and nine months of age. A copy of the completed Road-to-Health Card was filed in the maternal folder.

For this study, the growth outcomes, length and weight measurements at birth, three, six and nine months were used to estimate the weight-for-age (WAZ), length-for-age (LAZ) and weight-for-length (WLZ) z-scores using the WHO growth standards [19]. Infants were classified as being underweight, having stunting or wasting based on WAZ, LAZ and WLZ < -2 respectively [20]. Clinical outcomes included having a minimum of one episode of rash or skin infection, fever, upper respiratory tract infection (URTI), acute gastroenteritis (AGE) and lower respiratory tract infection (LRTI) between birth and nine months. Hospitalisation during the first nine months of infancy was also included as a clinical outcome.

Breastfeeding practice was ascertained by a questionnaire and documented as a “Yes” or “No” at three, six and nine months and duration of breastfeeding was determined at nine months or at the last clinic visit if infants were not seen at nine months. Infants in the HPTN046 study were tested for HIV at three, six and nine months using HIV DNA PCR assay. Mothers in the HPTN046 study had a CD4 count done at the time of infant enrolment.

Select data were extracted from the HPTN046 database and transferred to an excel spreadsheet. The substudy Investigator reviewed infant source documents from the CAP088 study and populated the excel spreadsheet with relevant data. The excel database was analysed using the SPSS version 24 statistical package. Descriptive statistics, such as frequencies and percentages, were used to summarise categorical data. We tested for normal data distribution using the Shapiro-Wilk test. With continuous variables that were not normally distributed, we report the median and IQR. Measures of central tendency mean and median and measures of dispersion such as standard deviation and interquartile range were calculated for numerical variables. Pearson chi-square test or Fisher’s exact test was used to test if there is an association between clinical and growth outcomes and breastfeeding duration in the two cohorts. An alpha value of 0.05 was considered significant.

## Results

Included in this secondary analysis were 123 HEU infants and 157 HU infants enrolled at birth. WLHIV and WNLHIV were similar in age, with a median age of 25 vs 23 years, respectively (Table 1). Majority of the women living with HIV or without HIV were not married and not living with their partner (91.8% vs 84.7%). A significantly higher proportion of women without HIV were employed (23.6% vs 4.9%) when compared to women living with HIV (*p* < 0.0001). WLHIV were generally healthy with a median CD4 count of 529 (IQR 457; 612) cells/ml and HEU and HU infants were of similar birth weight (3200 g) (*p* = 0.878). The two groups of infants (HEU and HU) differed significantly in the duration of breastfeeding, with a larger proportion of HU infants at a primary health care clinic breastfed for less than six months (52.9%) when compared to HEU infants in a breastfeeding study (38.2%) (*p* < 0.05) (Table 1). Overall, 70% of women who were employed versus 42% of unemployed breastfed for less than six months (*p* = 0.001).

**Table 1:** A comparison of Maternal and Birth Characteristics for Cohorts 1 and II

Characteristics	Cohort I: Infants Exposed to HIV (***n*** = 123)	Cohort II: Infants Not Exposed to HIV (***n*** = 157)	***P*** Value
Study Period	2007–2008	2017–2018	
Residence	Umlazi, South Africa	Umlazi, South Africa	
Maternal age (median [IQR])	25 [21; 29]	23 [19; 28]	0.072
**Relationship**			
Married	1 (0.8)	6 (3.8)	0.132
Not Married, Living with Partner	9 (7.4)	18 (11.5)	
Not Married, Not Living with Partner	112 (91.8)	133 (84.7)	
**Employed**			
No	116 (95.1)	120 (76.4)	< 0.0001
Yes	6 (4.9)	37 (23.6)	
**Mode of Delivery** [n (%)]			
Vaginal Delivery	87 (71.9)	126 (82.8)	0.039
Cesarean Section	34 (28.1)	26 (17.1)	
**Maternal CD4 count, cells/mm**^**3**^ (median [IQR])	529 [457; 612]	856 [706; 1044]	< 0.001
**Birth weight, g** [median IQR]	3200 [3000;3450]	3200 [2870; 3530]	0.373
**Birth Weight Category** [n (%)]			
< 2500 g	10 (7.6)	8 (5.4)	0.878
> 2500 g	121 (92.4)	140 (94.6)	
**Duration of Breastfeeding (weeks)**			
Median (IQR)	26 (25; 38)	14 (14; 38)	0.003
**Duration of breastfeeding Category** [n (%)]			
< 6 months	47 (38.2)	83 (52.9)	0.010
> 6 months	76 (61.8)	74 (47.1)	

Overall median WLZ scores were consistently and significantly lower in HEU than HU infants at three, six and nine months (Table 2) (− 0.19 vs 2.09; − 0.81 vs 0.28; 0.05 vs 0.97 respectively) (*p* < 0.001). The median WAZ score was significantly lower in HEU than HU infants at nine months only (0.41 vs 0.85; *p* < 0.05). In contrast, the median LAZ score was lower among HU infants at three, six and nine months (− 1.63 vs 0.91; − 0.37 vs 0.51; 0.13 vs 0.77) reaching statistical significance at three (*p* < 0.001) and six months (*p* < 0.05) only. When compared to HEU infants, a significantly larger proportion of HU infants were classified as underweight (WAZ < -2SD) at three months (1.6% vs 8.18%, *p* = 0.026) and stunted (LAZ < -2SD) at three and six months (3.9% vs 44.9, 4.8% vs 20.9% respectively) (*p* < 0.001).

**Table 2:** A comparison of Growth and Clinical Outcomes between Infants Exposed to and Not Exposed to HIV in the 1st nine months of life

Infant Growth and Clinical Outcomes	Infants Exposed to HIV	Infants Not Exposed to HIV	***P*** Value
**Infant Growth, WHO Z scores (median [IQR])**			
**Age 3 months**	*n* = 123	*n* = 107	
WAZ	0.27 (− 0.41; 0.88)	0.03 (− 0.69; 1.03)	0.360
LAZ	0.91 (− 0.24; 1.72)	− 1.63 (− 3.60; − 0.31)	< 0.001
WLZ	− 0.19 (− 1.26; 0.76)	2.09 (0.16; 3.79)	< 0.001
**Age 6 months**	n = 123	*n* = 67	
WAZ	0.31 (− 0.38; 0.85)	0.54 (− 0.40; 1.25)	0.115
LAZ	0.51 (− 0.48; 1.47)	− 0.37 (− 1.59; 0.99)	0.001
WLZ	0.17 (− 0.81; 0.79)	0.92 (0.28; 2.15)	< 0.001
**Age 9 months**	n = 123	*n* = 49	
WAZ	0.41 (−0.39; 0.97)	0.85 (− 0.03; 1.82)	0.007
LAZ	0.77 (− 0.22; 1.93)	0.13 (− 0.58; 1.37)	0.077
WLZ	0.05 (−0.67; 0.71)	0.97 (− 0.21; 2.15)	< 0.001
**Growth Faltering** ***n*** **(%)**			
**Age 3 months**			
Underweight	2 (1.6)	9 (8.18)	0.026
Stunting	5 (3.96)	48 (44.9)	< 0.001
Wasting	11 (8.73)	6 (5.61)	0.452
**Age 6 months**			
Underweight	4 (3.23)	1 (1.49)	0.659
Stunting	6 (4.84)	14 (20.9)	< 0.001
Wasting	9 (7.26)	3 (4.48)	0.545
**Age 9 months**			
Underweight	1 (0.82)	0	1.000
Stunting	7 (5.74)	1 (2.0)	0.440
Wasting	2 (1.64)	2 (4.0)	0.581
**Infants with 1 or more episodes** ***n*(%)**			
Rash/Skin Disease	59 (44.4)	20 (12.8)	< 0.0001
Fever	1 (0.8)	6 (3.9)	0.129
URTI	40 (30.1)	17 (10.9)	< 0.0001
Acute GE	7 (5.3)	5 (3.2)	0.395
LRTI	4 (3.0)	4 (2.6)	1.000

When compared to HU infants a markedly higher proportion of HEU infants experienced one or more episodes of skin rash (12.8% vs 44.5%) and upper respiratory tract infection (10.9% vs 30.1%) (*p* < 0.0001) (Table 2).

When stratified by breastfeeding duration, the growth and clinical outcomes among HU infants were not significantly different between infants breastfed for less than six months or six months or more (Table 3). In addition, the median LAZ score and the prevalence of stunting did not differ by breastfeeding duration among the HU infants (Table 4).

**Table 3:** A comparison of Growth and Clinical Outcomes between Infants Exposed to HIV and Breastfed for a minimum of 6 months or Breastfed for less than 6 months

Infant Growth and Clinical Outcomes	Breastfed **>** 6 months	Breastfed < 6 months	***P*** Value
**Infant Growth, WHO Z scores (median [IQR])**			
**Age 3 months**			
WAZ	0.40 (−0.23; 1.03)	−0.80 (− 0.65; 0.82)	0.051
LAZ	1.02 (− 0.09; 1.96)	0.54 (− 0.41; 1.49)	0.068
WLZ	−0.13 (−1.31; 0.58)	− 0.43 (−1.22; 0.82)	0.975
**Age 6 months**			
WAZ	0.32 (−0.17; 1.11)	0.20 (− 0.71; 0.53)	0.147
LAZ	0.61 (−0.64; 1.69)	0.46 (−0.33; 1.26)	0.878
WLZ	0.20 (−0.75; 1.06)	0.15 (−0.96; 0.62)	0.229
**Age 9 months**			
WAZ	0.47 (−0.35; 1.03)	0.29 (−0.62; 0.75)	0.488
LAZ	0.68 (−0.25; 1.45)	0.85 (−0.14; 1.98)	0.375
WLZ	0.11 (−0.51; 0.76)	0.09 (−1.08; 0.37)	0.117
**Growth Faltering** ***n*(%)**			
**Age 3 months**			
Underweight	1 (1.32)	1 (2.13)	0.620
Stunting	3 (3.95)	2 (4.26)	0.635
Wasting	6 (7.89)	5 (10.64)	0.416
**Age 6 months**			
Underweight	3 (3.95)	1 (2.17)	0.514
Stunting	3 (3.95)	2 (4.35)	0.626
Wasting	4 (5.26)	5 (10.87)	0.212
**Age 9 months**			
Underweight	1 (1.37)	0	0.624
Stunting	4 (5.48)	3 (6.82)	0.529
Wasting	1 (1.37)	1 (2.27)	0.613
**Infants with 1 or more episodes** ***n*(%)**			
Rash/Skin Disease	36 (47.37)	22 (46.81)	0.550
Fever	0	1 (2.13)	0.382
URTI	21 (27.63)	16 (34.04)	0.289
Acute GE	2 (2.63)	4 (8.51)	0.149
LRTI	1 (1.32)	2 (4.26)	0.325

**Table 4:** A comparison of Growth Outcomes between Infants Not Exposed to HIV and Breastfed for a minimum of 6 months or Breastfed for less than 6 months

Infant Growth and Clinical Outcomes	Breastfed **>** 6 months	Breastfed < 6 months	***P*** Value
**Infant Growth, WHO Z scores (median [IQR])**			
**Age 3 months**			
WAZ	0.17 (−0.31; 0.88)	−0.04 (−0.88; 1.13)	0.370
LAZ	−1.45 (−2.97; −0.31)	−1.77 (−3.69; − 0.01)	0.698
WLZ	2.09 (0.19; 3.99)	2.13 (0.16; 3.72)	0.839
**Age 6 months**			
WAZ	0.43 (−0.57; 1.24)	0.72 (−0.10; 1.71)	0.453
LAZ	−0.36 (−1.54; 0.85)	− 0.45 (− 2.07; 1.04)	0.978
WLZ	0.86 (− 0.06; 2.11)	1.56 (0.38); 2.75)	0.408
**Age 9 months**			
WAZ	0.81 (−0.02; 1.7)	1.22 (0.78; 2.14)	0.929
LAZ	−0.03 (− 0.75)	0.36 (− 0.23; 1.41)	0.280
WLZ	0.89 (0.13; 2.18)	1.31 (−0.63; 1.92)	0.812
**Growth Faltering** ***n*(%)**			
**Age 3 months**			
Underweight	0	8 (10.53)	0.058
Stunting	11 (37.93)	36 (48.0)	0.387
Wasting	1 (3.45)	5 (6.67)	0.463
**Age 6 months**			
Underweight	1 (2.08)	0	0.716
Stunting	9 (18.75)	5 (26.32)	0.353
Wasting	2 (4.17)	1 (5.26)	0.638
**Age 9 months**			
Underweight	0	0	
Stunting	1 (2.78)	0	0.720
Wasting	1 (2.78)	1 (7.14)	0.485

In a multivariable analysis, the odds of occurrence of wasting, skin rash, URTI or any clinical adverse event in HEU infants were 2.86, 7.06, 3.01 and 8.89 times higher than HU after adjusting for breastfeeding duration (Table 5).

**Table 5:** Multivariable Analysis of adverse growth outcomes and clinical adverse events in Infants Exposed to HIV relative to Infants Not Exposed to HIV adjusted for breastfeeding duration < 6 months

Infant Growth and Clinical Outcomes	Unadjusted OR (95%CI)	***P*** Value	Adjusted OR (95%CI)	***P*** Value
*Underweight at 3 or 6 months*				
Infants exposed to HIV	0.55 (1.84–1.66)	0.292	0.44 (0.11–1.79)	0.254
Infants not exposed to HIV	Ref		Ref	
*Stunting at 3 or 6 months*				
Infants exposed to HIV	0.15 (0.07–0.30)	< 0.001	0.15 (0.07–0.34)	< 0.001
Infants not exposed to HIV	Ref		Ref	
*Wasting at 3 or 6 months*				
Infants exposed to HIV	2.65 (1.10–6.36)	0.030	2.86 (1.09–7.50)	0.033
Infants not exposed to HIV	Ref		Ref	
*Rash/Skin Disease*				
Infants Exposed to HIV	5.42 (3.03–9.69)	< 0.001	7.06 (3.69–13.51)	< 0.001
Infants not exposed to HIV	Ref		Ref	
*Upper Respiratory Tract Infection*				
Infants Exposed to HIV	3.52 (1.88–6.57)	< 0.001	3.01 (1.50–6.03)	0.002
Infants not exposed to HIV	Ref		Ref	
*Any Clinical Adverse Event*				
HIV Exposed Uninfected	6.35 (3.78–10.68)	< 0.001	8.89 (4.75–16.61)	< 0.001
Breastfeeding< 6 months	Ref		Ref	

## Discussion

In this secondary analysis of growth, clinical and breastfeeding data collected for HEU and HU infants, we report a significantly shorter breastfeeding duration (median 14 weeks) and a higher occurrence of stunting (45% at 3 months and 21% at 6 months) among HU infants in the first six months of life. The shorter duration of breastfeeding was not associated with stunting. We also report lower WLZ scores and higher frequency of rash/skin disease and URTI among the HEU infants independent of breastfeeding duration.

Breastfeeding has long been established as the best form of adequate nutrition in infants to reduce the risk of childhood morbidity and mortality [9, 10]. The WHO has made large investments towards promoting exclusive breastfeeding for six months and continued breastfeeding up to two years [11]. In our study, the median duration of breastfeeding was 20 weeks among HU infants and 26 weeks among HEU infants. Only 46% of HU and 61% of HEU infants were breastfed for six months or more. Early cessation of breastfeeding in the South African population has also been reported in other studies independent of HIV exposure [13, 14]. In Horwood’s study, mothers who were returning to work or school were less likely to breastfeed (AOR 3.76) [14]. This is consistent with our findings: 70% of women who were employed breastfed for < 6 months versus 42% of unemployed breastfed < 6 months.

In our study, a significantly higher proportion of HU infants was underweight at three months (8.2% vs 1.6%) and stunted at three and six months (44.9% vs 3.96%; 20.9% vs 4.84%) when compared to their HEU counterparts. Other South African studies have also reported a high prevalence of stunting in children (28.5%) [21, 22]. Although a larger proportion of the HU infants were breastfed for less than six months, stunting and being underweight were independent of breastfeeding duration. The occurrence of stunting even among the longer breastfed infants is suggestive of other factors that could contribute to the high prevalence of stunting such as mixed feeding or poor quality of breastmilk as a result of poor nutrition in lactating mothers [23, 24]. More recent studies have underscored the role of maternal nutrition among lactating mothers [25, 26]. Maternal nutrition supplementation preconception or early pregnancy was shown to improve linear growth in infants in the first six months, suggesting that poor nutrition in lactating mothers could influence infant growth despite optimal breastfeeding practice [25, 26].

We report significantly lower median WLZ scores among HEU infants at three, six and nine months and WAZ score at nine months in comparison to HU infants and independent of breastfeeding duration. Poor growth has long been associated with HIV exposure; however, nutrition and socioeconomic status have also been considered as significant determinants. In a cross-sectional study in Botswana where HEU infants were more likely to have been formula-fed, a higher proportion of HEU infants between six and 24 months was underweight and stunted underscoring the benefits of breastfeeding [27]. However, even if breastfeeding is the norm, in the slums of Nairobi, Kenya stunting was the most common form of undernutrition among HEU infants when compared to their HU counterparts [28]. The authors concluded that high undernutrition among HEU infants was as a result of HIV exposure, the number of children in a household and the lack of food aid [28]. Here again, we raise the question of nutrition among lactating mothers. Consistent with our findings, a Ugandan study conducted in the pre-ART era also concluded that duration of breastfeeding was not associated with adverse growth outcomes in HEU infants [29]. No matter how long women breastfed, if nutritionally compromised themselves, they are more likely to provide inadequate nutrition to their infants via breastfeeding [25, 26].

After adjusting for breastfeeding duration, we have shown that HEU infants were 2.9, 7.1, 3.0 and 8.9 times at risk of wasting, skin rash, URTI, or any clinical adverse event respectively when compared to their HU counterparts. Consistent with other studies in the pre-ART era, HIV exposure was independently associated with a higher frequency of any clinical event in early infancy. The largest HIV-exposed, uninfected cohort (ZVITAMBO), which prospectively followed up 14 110 infants in Zimbabwe before the availability of ART, reported higher morbidity and three times higher mortality in HEU infants when compared to HU infants; this mortality risk was higher in the first year of life compared with the second [30]. A study in South Africa after universal ART became available, reported significantly more hospitalisations, five times higher prevalence of lower respiratory tract infections and three times higher diarrhoeal diseases in HEU infants compared to HU infants (*n* = 410) [31]. The higher incidence was attributed to advanced maternal HIV disease and late ART initiation.

## Conclusion

Our study has generated additional evidence that infants exposed to HIV are at substantial risk of infectious morbidity and decreased growth trajectories independent of breastfeeding duration. We further report shorter breastfeeding duration in infants not exposed to HIV. And the higher prevalence of stunting in this cohort is also independent of breastfeeding duration.

### Limitations

Our study is not without limitations. We accept that non-contemporaneous studies are not ideal, but this was the only opportunity to disentangle the effect of ART from HIV on infant growth outcomes. To reduce the selection bias, we selected the HEU cohort specifically enrolled from the same community as the HU cohort but only a few years apart. There is much evidence supporting the association between HIV infection and preterm birth, and in addition infants born preterm are also more likely to have poor growth trajectories regardless of birthweight. Unfortunately, we were unable to adjust our analysis for preterm births as gestational age data were not collected.

The high attrition rate of HU infants at three (32%), six (57%) and nine (69%) months was not unusual at the primary health clinic. As a result, we acknowledge that our findings at nine months are not conclusive due to the small number of HU infants assessed at this time point. Another limitation to our study findings is the lack of breastfeeding quality data and maternal nutritional status.

### Implications of our findings

The findings presented in this study raise concerns on maternal nutrition irrespective of HIV exposure in SSA. The possible interplay of maternal nutrition, the role of maternal nutrition supplements preconception or in early pregnancy must be considered when developing breastfeeding policies for HIV unexposed, and HIV exposed mother-infant pairs. There is a need for other studies exploring maternal nutrition in association with breastmilk content and infant clinical and growth outcomes.

## Data Availability

The data that support the findings of this study are available from the corresponding author, upon reasonable request.

## Abbreviations

HIV: Human Immunodeficiency Virus
LMIC: Low and Middle Income Country
HEU: HIV Exposed Uninfected
HU: HIV Unexposed
WLZ: Weight-for-length Z score
LAZ: Length-for-age Z score
WAZ: Weight-for-age Z score
AGE: Acute gastroenteritis
URTI: Upper respiratory tract infection
LRTI: Lower respiratory tract infection
SSA: Sub Saharan Africa
SA: South Africa
PMTCT: Prevention of mother-to-child transmission
cART: Combination antiretroviral treatment
WLHIV: Women living with HIV
WNLHIV: Women not living with HIV
NVP: Nevirapine
